# Gut–Liver Axis Mechanisms Underlying Spontaneous Reversal of Liver Fibrosis: A Gut Microbiota-Metabolomics Analysis

**DOI:** 10.3390/metabo16060424

**Published:** 2026-06-17

**Authors:** Yuanying Zhao, Hao Chang, Chenxue Hou, Bingqing Yang, Yue Li

**Affiliations:** 1 Department of Clinical Laboratory, Beijing Ditan Hospital, Capital Medical University, Beijing 100015, China; zhaoyuanying0804@163.com (Y.Z.); hcx20010123@163.com (C.H.); 2 Center of Liver Diseases, Peking University Ditan Teaching Hospital, Beijing 100015, China; 18994726606@163.com; 3 Center of Liver Diseases, Beijing Ditan Hospital, Capital Medical University, Beijing 100015, China; ybq1593572022@163.com

**Keywords:** liver fibrosis reversal, untargeted metabolomics, 16S rRNA, gut–liver axis

## Abstract

**Background:** The reversal of liver fibrosis is crucial for improving outcomes in chronic liver disease. The gut–liver axis, mediated by the intestinal microbiota, plays a significant role in this process. However, its dynamic changes and mechanisms during reversal remain unclear. This study aimed to systematically reveal these dynamics and explore the link between gut microbiota and metabolism in a spontaneous reversal model. **Methods:** Intestinal contents were collected from mouse model groups (fibrosis, 4-week reversal, and 12-week reversal). The use of 16S rRNA gene sequencing was employed to analyze gut microbiota structure, and untargeted metabolomics was used to profile metabolic changes. Differential metabolites and microbial taxa were identified using multivariate statistical analysis, followed by pathway enrichment analysis. Spearman correlation analysis was used to construct metabolite–microbiota association networks across different reversal stages. **Results:** Metabolomic analysis showed significant alterations in multiple pathways during reversal. Linoleic and α-linolenic acid metabolism had a high impact in later stages. Taurine and biotin metabolism remained active throughout. Branched-chain amino acid degradation was enriched later. Microbiota analysis revealed significant structural shifts via beta-diversity. Bacteroidota decreased while Firmicutes increased in 4 weeks. Butyrate-producing families increased, and Akkermansia was enriched later. Integrated analysis demonstrated significant correlations between specific bacteria and metabolites, indicating a close microbiota–metabolism association during reversal. **Conclusions:** This integrated multi-omics study delineates the potential dynamic reorganization of the gut microbiota and host metabolism during spontaneous liver fibrosis reversal. These findings provide a theoretical basis for understanding the gut–liver axis mechanism in fibrosis reversal and for developing microbiota-targeted intervention strategies.

## 1. Introduction

Liver fibrosis, as the core pathological process in the progression of chronic liver disease, represents an abnormal repair response following liver injury caused by various factors such as viral infections and metabolic abnormalities. It is characterized by an imbalance between the synthesis and degradation of the extracellular matrix (ECM) within the liver, leading to the accumulation of extensive abnormal scar tissue [1,2]. Liver fibrosis, as a key pathological step in the progression of chronic liver disease, is not an independent disease entity. When the liver sustains persistent injury, the fibrotic process progressively worsens. Without effective intervention, it may ultimately progress to cirrhosis or even induce hepatocellular carcinoma [3], posing a serious threat to human health. Traditionally, liver fibrosis was considered an irreversible process. However, recent clinical observations and basic research have demonstrated that after eliminating or effectively controlling the causative factors, liver fibrosis can exhibit varying degrees of reversal [4,5]. This discovery offers renewed hope for liver disease treatments. Although significant progress has been made in research on reversing liver fibrosis, some underlying molecular mechanisms remain incompletely elucidated. Therefore, systematically clarifying the molecular mechanisms driving fibrosis reversal holds significant theoretical and practical value for advancing the diagnosis and treatment of liver fibrosis.

During the onset and progression of liver fibrosis, metabolic reprogramming is considered one of the core pathophysiological features. As the body’s metabolic hub, the liver undergoes significant alterations in metabolic patterns within hepatocytes, immune cells, and activated hepatic stellate cells when subjected to persistent injury. Studies indicate that amino acid and nucleotide/glycolytic pathways become markedly disrupted in fibrotic models [6]. Reversing such metabolic dysregulation can effectively mitigate the progression of fibrosis. Furthermore, abnormalities in bile acid metabolism are also closely associated with liver fibrosis [7,8,9]. These findings suggest that restoring metabolic homeostasis may be a critical step in reversing liver fibrosis, and metabolomics offers a novel perspective for understanding the mechanisms of fibrosis reversal at the host metabolic level.

Meanwhile, with the advancement of high-throughput sequencing technology, 16S rRNA gene sequencing has been widely applied in liver fibrosis research to analyze gut microbiota composition. Studies have revealed that in non-obese patients with non-alcoholic fatty liver disease (NAFLD), changes in the abundance of Ruminellaceae and Verrezonellaceae are significantly correlated with the severity of liver fibrosis [10]. Another study demonstrated that Akkermansia species abundance declines in animal models of liver fibrosis [11]. Following the intervention, the restoration of this abundance was closely associated with improvements in liver fibrosis. These findings suggest that specific gut microbial groups may serve as potential biomarkers for reversing liver fibrosis.

Currently, few studies have systematically analyzed the dynamic changes and interactions between metabolites and gut microbiota during the spontaneous reversal of liver fibrosis. Therefore, this study utilizes an established spontaneous reversal model of liver fibrosis in mice developed by our research group. By integrating 16S rRNA gene sequencing with non-targeted metabolomics technology to analyze mouse intestinal contents, we aim to systematically elucidate the response patterns of metabolites and liver repair-related metabolic pathways during liver fibrosis reversal, the dynamic evolution of gut microbiota structure, and the intrinsic relationship between gut microbiota reprogramming and metabolic reprogramming during liver fibrosis reversal. This study aims to provide new theoretical insights into the mechanisms of liver fibrosis reversal through multi-omics integration analysis and identify potential targets for developing anti-fibrotic strategies based on the regulation of the liver–gut axis.

## 2. Materials and Methods

### 2.1. Based on LC-MS Untargeted Metabolomics Analysis

Metabolomic sequencing analysis was performed on the intestinal contents of mice from our previously established CCl_4_-induced liver fibrosis reversal model, with three biological replicates per group (*n* = 3 per group for the fibrosis model, the 4-week reversal group, and the 12-week reversal group). The detailed characterization of progressive fibrosis regression at each reversal stage, confirmed by histological and molecular analyses, has been described in our previous study [12]. All sample processing and mass spectrometry detection were conducted by Biotech-Pack Scientific (Shanghai, China). Weigh approximately 25 mg of sample and add 500 μL of extraction solution pre-chilled to −40 °C. Add steel beads and homogenize the sample at 35 Hz for 4 min. Sonicate in an ice-water bath for 5 min. Repeat this procedure three times. Incubate at 40 °C for 1 h. Transfer 300 μL to a 96-well filter plate. Place the filter plate-collection plate assembly into a positive-pressure device. Slowly pressurize to 6 psi and maintain for 3 min. Remove the filter plate-collection plate assembly from the device. Prepare an equal volume of supernatant from another sample to create a quality control (QC) sample for testing.

For polar metabolites, this project employs a Vanquish (Thermo Fisher Scientific, Waltham, MA, USA) ultra-high-performance liquid chromatography (UPLC) system. Target compounds are chromatographically separated using a Waters ACQUITY UPLC BEH Amide column (2.1 mm × 100 mm, 1.7 μm). Liquid chromatography (LC) mobile phase A consists of an aqueous solution containing 25 mmol/L ammonium acetate and 25 mmol/L ammonium hydroxide, while phase B comprises acetonitrile. Sample tray temperature: 4 °C. Injection volume: 2 μL.

For non-polar metabolites, this project employs a Vanquish ultra-high-performance liquid chromatography system (Thermo Fisher Scientific, Waltham, MA, USA) to chromatographically separate target compounds using a Phenomenex Kinetex C18 column (2.1 mm × 100 mm, 2.6 μm). LC mobile phase A consists of an aqueous solution containing 0.01% acetic acid, while phase B comprises isopropanol–acetonitrile (1:1, *v*/*v*). Sample tray temperature: 4 °C. Injection volume: 2 μL.

Primary and secondary mass spectrometry data were acquired using an Orbitrap Exploris 120 mass spectrometer controlled by Xcalibur software (version 4.4, Thermo). Metabolite identification was performed using an R package developed in collaboration with Group 7, employing the BiotreeDB (V3.0) database, followed by visualization analysis using a custom-built R package.

Data were analyzed using principal component analysis (PCA) and orthogonal partial least squares discriminant analysis (OPLS-DA) modeling with SIMCA software (V18.0.1, Sartorius Stedim Data Analytics AB, Umea, Sweden). Identified metabolites were annotated using databases such as KEGG and PubChem. Heatmaps were generated using the R package R (pheatmap), while metabolic pathway bubble plots were created using R (KEGGgraph, ggplot2).

### 2.2. 16S rRNA Gene Sequencing and Microbial Diversity Analysis

The same intestinal content samples used for metabolomics analysis were also subjected to 16S rRNA gene sequencing. Microorganisms from intestinal content samples were extracted using the OMEGASoil DNA Kit (D5635-02) (OmegaBio-Tek, Norcross, GA, USA) according to the kit instructions. DNA fragment sizes were analyzed by 0.8% agarose gel electrophoresis, and DNA concentrations were measured using the Nanodrop2000 (ThermoFisher Scientific, Inc., USA). Amplify the V3-V4 region of the bacterial 16S rRNA gene using forward primer 5′-ACTCCTACGGGAGGCAGCA-3′ and reverse primer 5′-GGACTACHVGGGTWTCTAAT-3′. PCR amplification was performed using Pfu ultra-high fidelity DNA polymerase. For thermal cycling, the initial step involved pre-denaturation at 98 °C for 5 min, followed by cycles of denaturation at 98 °C for 30 s, annealing at 53 °C for 30 s, and extension at 72 °C for 45 s, repeated 28 times. The reaction concluded with a final extension at 72 °C for 5 min. The size of the amplified target band was detected using 1% agarose gel electrophoresis to confirm that the fragment matched the expected size. Subsequently, the PCR product was purified using the Agencourt AMPure XP™ Nucleic Acid Purification Kit (Beckman Coulter, Inc., Brea, CA, USA).

Quantification of purified PCR products was performed using the Quant-iT picoGreen dsDNA Assay Kit. Samples were pooled according to the required data volume per sample for library preparation. Library construction was carried out using the NEBNext Ultra II DNA Library Prep Kit (New England Biolabs, Inc., Ipswich, MA, USA). Sequencing was performed using the Illumina NovaSeq 6000 platform (Illumina, Inc., San Diego, CA, USA) with 2 × 250 bp paired-end reads. All experiments were completed at Baiqu Biomedical Technology Co., Ltd. (Shanghai, China).

Filter the top 50 ASV sequences by abundance and construct species evolutionary trees for annotated ASVs using the “phylogenyalign-to-tree-mafft-fasttree” workflow in QIIME2 software (2024.2). Perform rarefying using the qiime feature-table rarefy function in QIIME2, randomly sampling the same number of sequences (i.e., the minimum sequence count across all samples) from each sample as the standard; calculate relative abundance tables after rarefying. Perform alpha and beta diversity analyses using QIIME2 software. Employ the species-level relative abundance table for LefSe analysis with LDA > 3.0 and *p* < 0.05 as screening thresholds, conducting pairwise or multi-group comparisons. Using the trimmed ASV abundance table and ASV sequence data, perform functional prediction analysis with PICRUST2 (v2.5.2) software and generate KEGG abundance bar charts using Python (3.10.12) scripts.

### 2.3. Statistical Analysis

Metabolomics data were normalized prior to statistical analysis, and the normalized data were assumed to approximate a normal distribution. Differential metabolites were identified using orthogonal partial least-squares discriminant analysis (OPLS-DA) with variable importance in projection (VIP) > 1.0, combined with Student’s *t*-test *p* < 0.05. False discovery rate (FDR) correction was applied using the Benjamini–Hochberg method, and the resulting q-values are reported. OPLS-DA models were validated using 7-fold cross-validation and 200 rounds of permutation testing. Identification confidence was annotated according to the Metabolomics Standards Initiative (MSI) criteria: Level 1, matching MS1, MS2, and RT with an authentic standard; Level 2, matching MS1 and MS2 with public databases; Level 3, matching MS1, MS2, and predicted RT with theoretical databases; Level 4, unknown compounds. Only metabolites detected in at least 80% of samples within any group were retained for further analysis. For microbiota data, differentially abundant taxa were identified using LEfSe with default significance thresholds.

## 3. Results

### 3.1. Changes in Gut Metabolites in Mice During the Reversal of Liver Fibrosis

**Figure 1:**
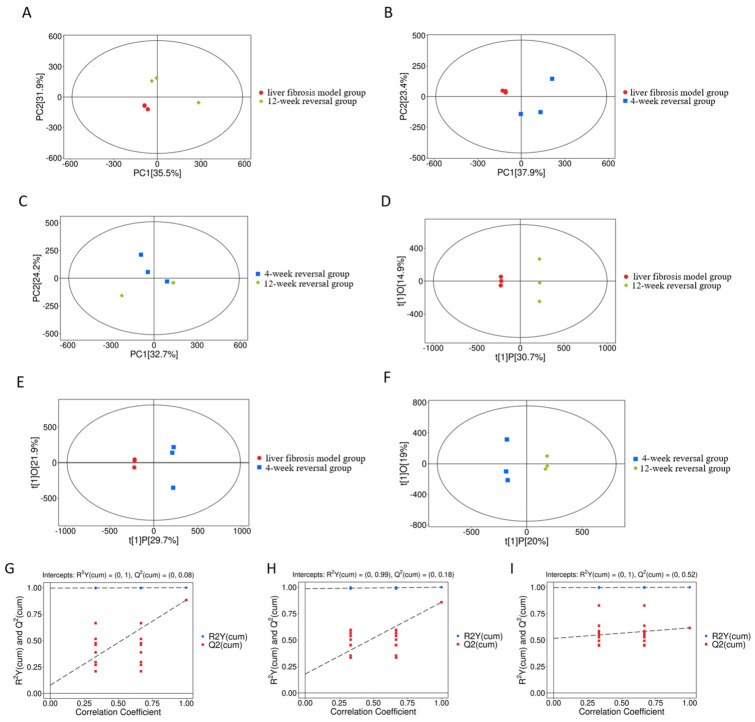
Identification of differences in gut metabolite levels among groups via PCA and OPLS-DA analysis. (**A**) PCA plot of the liver fibrosis model group vs. the 12-week reversal group. (**B**) PCA plot of liver fibrosis model group vs. 4-week reversal group. (**C**) PCA plot of the 4-week reversal group vs. the 12-week reversal group. (**D**) OPLS-DA score plot of liver fibrosis model group vs. 12-week reversal group. (**E**) OPLS-DA score plot of liver fibrosis model group vs. 4-week reversal group. (**F**) OPLS-DA score plot of the 4-week reversal group vs. the 12-week reversal group. (**G**–**I**) Validation plot obtained from 200 permutation tests, respectively.

To investigate the effects of this pathway for reversing liver fibrosis on intestinal metabolites in mice, metabolomic analysis was performed on intestinal contents from mice in the liver fibrosis model group, the 4-week reversal group, and the 12-week reversal group. A total of 7764 gut metabolites were identified, primarily including linoleic acid and its derivatives, bile acids, alcohols and their derivatives, fatty acids and polymers, amino acids, peptides and analogs, carbohydrates and carbohydrate conjugates, among others. To determine differences in gut metabolite levels among groups, principal component analysis (PCA) and orthogonal partial least squares discriminant analysis (OPLS-DA) were performed. PCA results showed a distinct separation between the liver fibrosis model group and the 12-week reversal group along principal components (PC1: 35.5%, PC2: 31.9%, Figure 1A). Simultaneously, separation was observed between the model group and the 4-week reversal group (PC1: 37.9%, PC2: 23.4%, Figure 1B). However, the 4-week reversal group and the 12-week reversal group were not completely separated (PC1: 32.7%, PC2: 24.2%, Figure 1C). Based on the OPLS-DA model, we aimed to identify genuine intergroup differences and screen for effective differential metabolites. Results showed strong discrimination between the model group and the 12-week reversal group (R^2^Y = 1, Q^2^ = 0.883, Figure 1D,G) and between the model group and the 4-week reversal group (R^2^Y = 1, Q^2^ = 0.858, Figure 1E,H). However, the 4-week reversal group and the 12-week reversal group were not well distinguished (R^2^Y = 0.999, Q^2^ = 0.615, Figure 1F,I).

### 3.2. Metabolite and Pathway Enrichment Analysis Reveals Dynamic Changes During the Reversal of Liver Fibrosis

**Figure 2:**
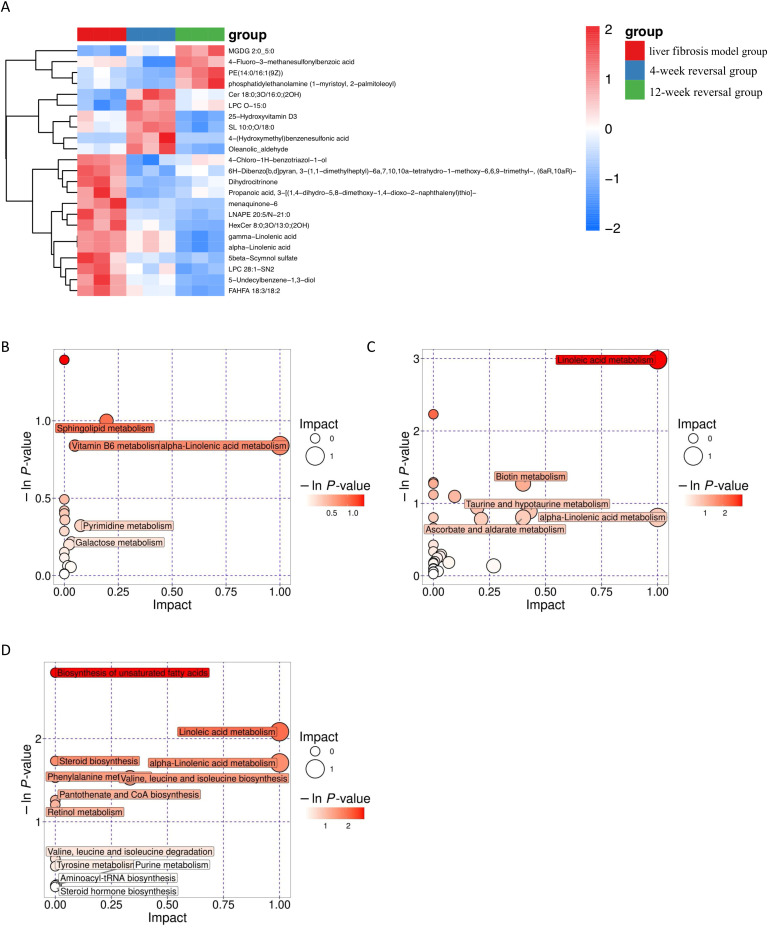
Analysis of differentially metabolized compounds in gut content samples across three groups. (**A**) The hierarchical clustering results for differential metabolites. (**B**) Functional enrichment analysis of differentially expressed metabolites between the liver fibrosis model group and the 4-week reversal group. (**C**) Functional enrichment analysis of differentially expressed metabolites between the liver fibrosis model group and the 12-week reversal group. (**D**) Functional enrichment analysis of differentially expressed metabolites between the 4-week reversal group and the 12-week reversal group.

To further identify key metabolites involved in the spontaneous reversal of liver fibrosis, we performed non-targeted metabolomics analysis on intestinal contents from three groups of mice and conducted hierarchical clustering analysis. Using an OPLS-DA VIP score > 1.0 as the threshold and a *p*-value < 0.05 for the fold change in expression levels between any two groups (liver fibrosis model group, 4-week reversal group, and 12-week reversal group) as the criterion, a total of 23 differentially metabolized products were identified, as shown in Supplementary Table S1. According to the Metabolomics Standards Initiative (MSI) criteria, the majority of these metabolites were classified as Level 2, while a small number reached Level 1. We observed that γ-linolenic acid and α-linolenic acid exhibited the highest expression levels in the model group, remained elevated in the spontaneous reversal 4-week group, and decreased significantly in the reversal 12-week group. This trend may correlate with their involvement in the linoleic acid/α-linolenic acid metabolic pathway during reversal. Concurrently, the fatty acid ester product FAHFA (18:3/18:2) exhibited the highest expression in the model group and showed a gradual decline during reversal, suggesting that this class of anti-inflammatory lipid molecules may be compensatorily elevated during the fibrotic phase and gradually return to normal levels during reversal. Heatmap analysis further revealed that 25-hydroxyvitamin D3 exhibited the lowest expression in the model group, significantly increased in the 4-week reversal group, and slightly decreased in the 12-week reversal group. Conversely, menaquinone-6 (a form of vitamin K2) showed the highest expression in the liver fibrosis model group and exhibited a sustained decline during reversal. Regarding lipids, LPC 28:1-SN2 and HexCer (8:0;3O/13:0;(2OH)) exhibited higher expression in the model group and decreased expression in the reversal groups. Additionally, several specific metabolites with potential biological activity showed significant changes, such as PE(14:0/16:1(9Z)) and 5β-Scymnol sulfate (Figure 2A).

To systematically elucidate the metabolic mechanisms underlying the spontaneous reversal of liver fibrosis, pathway enrichment analysis was performed on mouse intestinal contents. Metabolic reprogramming is initiated early in the reversal process. The sphingolipid metabolism pathway exhibited high significance, suggesting its potential involvement in the reversal process. Concurrently, vitamin B6 metabolism and α-linolenic acid metabolism also showed significant enrichment (Figure 2B). Compared to the model group, after 12 weeks of reversal, linoleic acid metabolism and α-linolenic acid metabolism reappeared with higher impact values, confirming the sustained role of polyunsaturated fatty acid metabolism throughout the reversal process. Furthermore, enrichment in taurine and hyportaurine metabolism, biotin metabolism, as well as ascorbic acid and aldonic acid metabolism highlighted the importance of antioxidant stress and energy metabolism in long-term reversal (Figure 2C). As shown in Figure 2D, the activation of unsaturated fatty acid biosynthesis, steroid biosynthesis, and the degradation pathways of valine, leucine, and isoleucine provides the material and energy foundation for hepatocyte regeneration and functional recovery.

### 3.3. Changes in Gut Microbiota Community Structure During the Reversal of Liver Fibrosis

**Figure 3:**
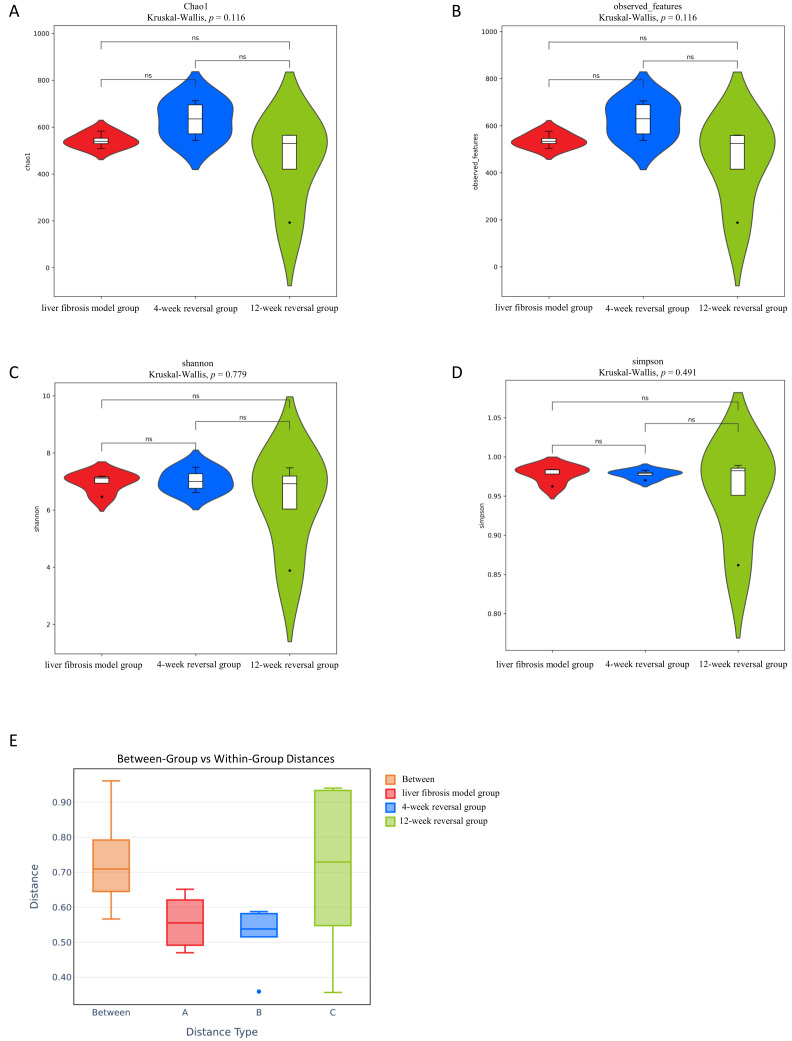
Analysis of α-diversity and β-diversity of the microbiota during liver fibrosis reversal. (**A**–**D**) Alpha-diversity of the bacterial community was measured using Chao1 (**A**), Observed_features (**B**), Shannon (**C**), and Simpson (**D**). (**E**) ANOSIM analysis of differences in community composition between groups. Each dot represents an individual sample. Points below the lower quartile or above the upper quartile are shown as individual dots.

To evaluate changes in gut microbiota structure during liver fibrosis and its spontaneous reversal, we performed 16S rRNA gene sequencing on intestinal contents from mice in the model group, the 4-week reversal group, and the 12-week reversal group, analyzing their α and β-diversity. Analysis of α-diversity indicated that the overall community structure remained stable across groups. As shown in Figure 3A,B, the Chao1 index (*p* = 0.116) and Observed_features index (*p* = 0.116), which characterize community richness, showed no significant differences among the three groups. Similarly, as depicted in Figure 3C,D, the Shannon index (*p* = 0.779) and Simpson index (*p* = 0.491), which characterize community diversity and evenness, also showed no significant changes across the three groups. However, β-diversity analysis indicated differences in community structure between groups, suggesting a preliminary trend toward separation in microbial community composition among Groups A, B, and C (Figure 3E). Analysis of similarities (ANOSIM) revealed strong community separation between the model group and the 4-week reversal group (R = 0.979, *p* = 0.031). Significant community differences were also observed between the model group and the 12-week reversal group (R = 0.552, *p* = 0.031), as well as between the 4-week reversal group and the 12-week reversal group (R = 0.469, *p* = 0.034) (Supplementary Table S2). This suggests that gut microbiota composition continues to evolve at different stages of the reversal process.

### 3.4. Dynamic Evolution of Gut Microbiota Community Composition During Liver Fibrosis Reversal

**Figure 4:**
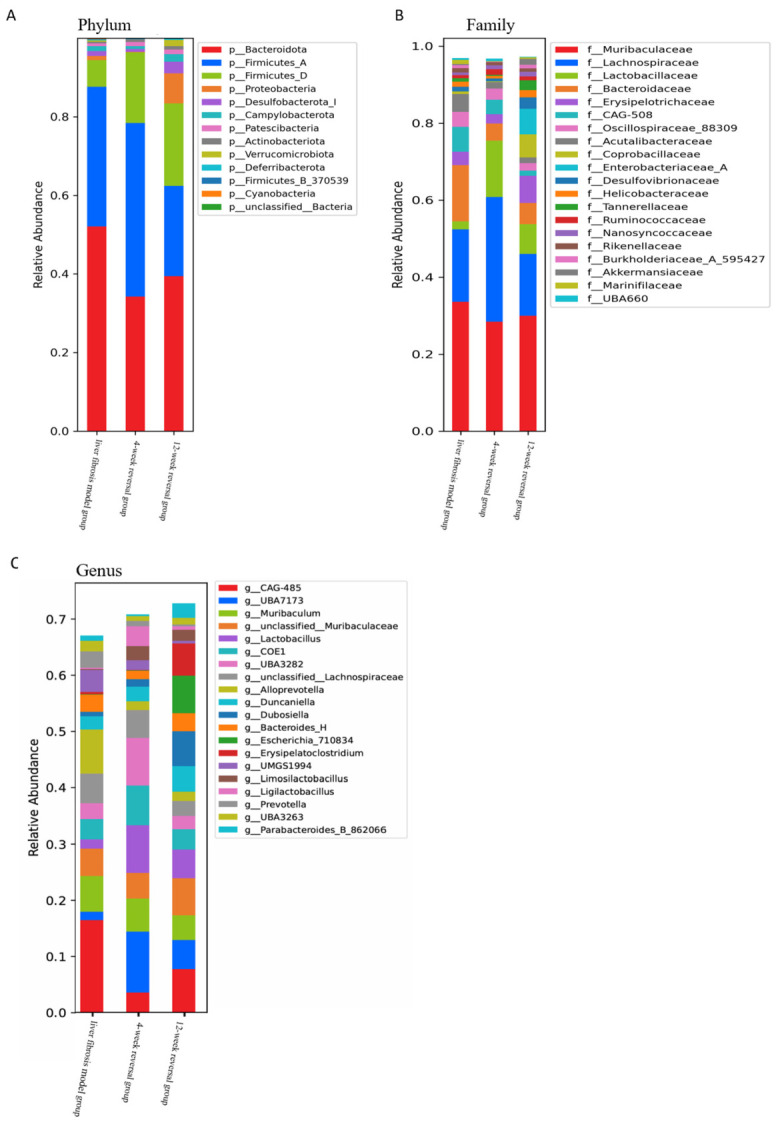
Changes in gut microbiota community composition across three groups. (**A**) Relative abundance of microbial communities at the phylum level across groups. (**B**) Relative abundance of microbial communities at the family level across groups. (**C**) Relative abundance of microbial communities at the genus level across groups.

Based on the confirmation of significant differences in community structure, we further analyzed the relative abundance of microbial communities at different levels. At the phylum level, the overall community pattern remained stable, but the proportions of key phyla changed. As shown in Figure 4A, the gut microbiota of all groups were dominated by the Bacteroidetes phylum and the Firmicutes phylum. However, compared to the model group, the 4‑week reversal group exhibited a trend of decreased relative abundance of Bacteroidetes and increased relative abundance of Firmicutes, resulting in an upward trend in the Firmicutes/Bacteroidetes (F/B) ratio during early reversal phase. At the family and genus levels, changes in functionally specific microbiota were more pronounced, revealing the direction of microbial reshaping during reversal. The relative abundance of Akkermansia was significantly higher in the 12-week reversal group than in the model group and the 4-week reversal group (Supplementary Table S3). Families associated with short-chain fatty acid production: As shown in Figure 4B, important butyrate-producing families such as Lachnospiraceae and Ruminococcaceae exhibited increasing trends in relative abundance in the 4-week reversal group. The Muribaculaceae family, one of the core gut microbiota, also exhibited dynamic changes in relative abundance across groups, potentially participating in the liver–gut axis process through its bile acid hydrolase activity. Compared with the control group, genera typically considered beneficial, such as Lactobacillus, exhibited more stable and positive growth trends in relative abundance across reversal groups (Figure 4C). 

### 3.5. Dynamic Evolution of Gut Microbiota KEGG Functional Profiles During Liver Fibrosis Reversal

To systematically analyze the evolution of the functional potential of the gut microbiota during the reversal of liver fibrosis, we used 16S rRNA sequencing data and the PICRUSt2 tool to predict the KEGG-based functional profiles of the microbial community.

**Figure 5:**
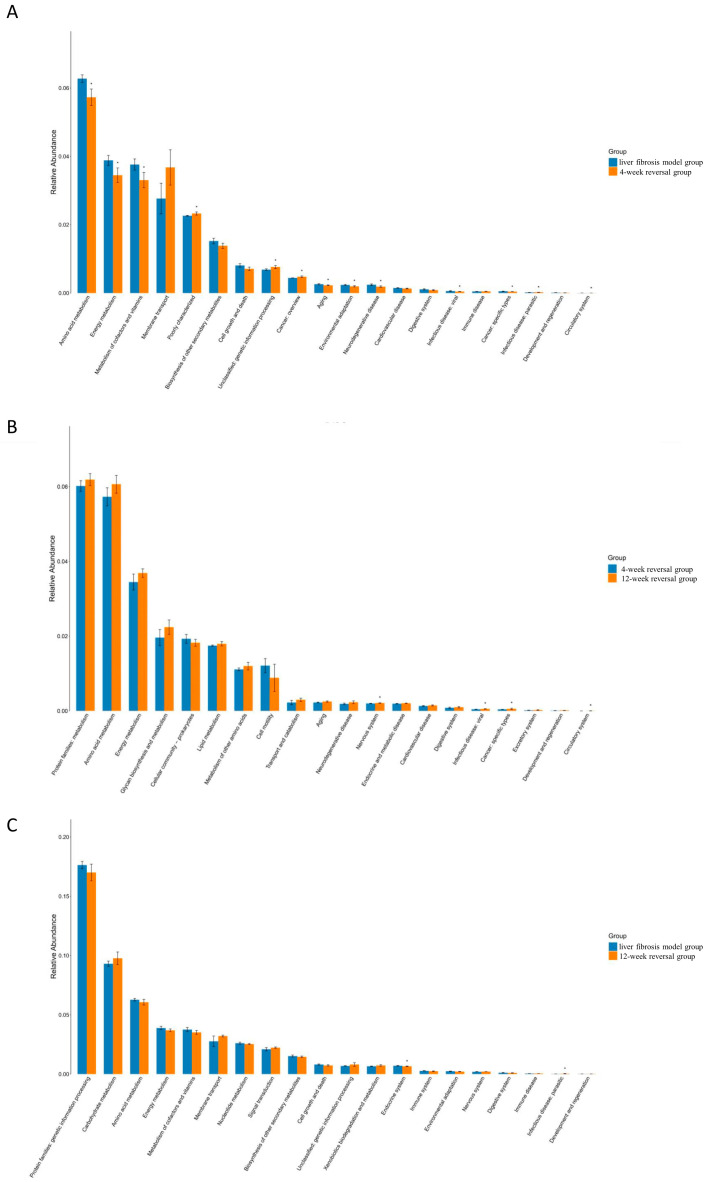
Differential enrichment of gut microbiota KEGG functional profiles. (**A**) KEGG pathways between the liver fibrosis model group and the 4-week reversal group. (**B**) KEGG pathways between the 4-week reversal group and the 12-week reversal group. (**C**) KEGG pathways between the liver fibrosis model group and the 12-week reversal group (* *p* < 0.05).

During the early reversal phase, the predicted functional profile of the gut microbiota—derived from PICRUSt2 inference based on 16S rRNA gene data—underwent significant restructuring. Compared to the model group, the 4-week reversal group exhibited the most pronounced enrichment in microbial gene families associated with amino acid metabolism, energy metabolism, and coenzyme and vitamin metabolism. These predicted changes suggest that early in reversal, gut microbes may enhance core metabolic functions related to basal synthesis and energy supply, potentially contributing to the initial metabolic momentum of the reversal process (Figure 5A). When comparing the 4-week and 12-week reversal groups, the predicted functional differences were primarily enriched in pathways related to the nervous system, infectious diseases (viral), cancers (specific types), and the circulatory system (Figure 5B). This indicates that the functional shift during the later phase of reversal may involve host-microbe interactions associated with immune modulation and systemic physiological regulation. Comparison between the model group and the 12-week reversal group showed that the predicted functional profiles were mainly distinguished by enrichment in endocrine system pathways and infectious diseases (parasitic) (Figure 5C).

### 3.6. Correlation Between Gut Microbiota and Metabolic Products

**Figure 6:**
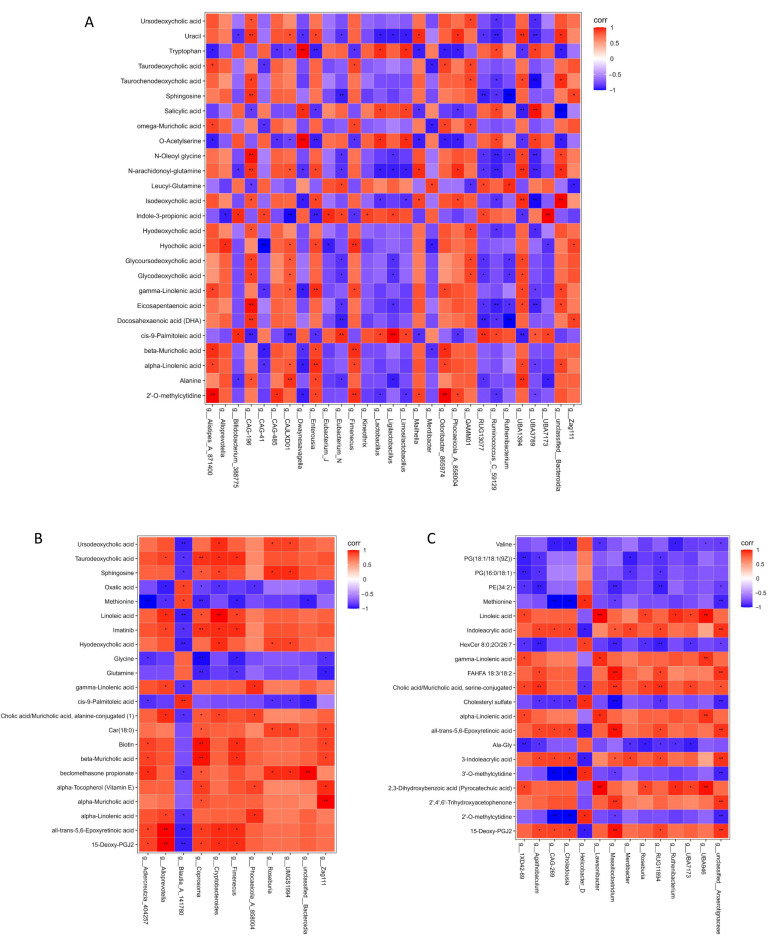
Correlation analysis between microbiota and differentially expressed metabolites. (**A**) Fibrosis model group vs. 4-week reversal group. (**B**) Fibrosis model group vs. 12-week reversal group. (**C**) 4-week reversal group vs. 12-week reversal group. (* *p* < 0.05, ** *p* < 0.01.)

To investigate the co-evolution patterns of metabolites and gut microbiota during liver fibrosis reversal, a correlation network was constructed using Spearman’s correlation analysis. This identified key metabolite–microbiota pairs exhibiting significant shifts during reversal, providing evidence for elucidating the role of the gut–liver axis in fibrosis reversal. Results from the model group and the 4-week reversal group showed that cis-9-Palmitoleic acid exhibited a strong correlation with Eubacterium (r = 0.941, *p* = 0.0051) and a highly positive correlation with Ruminococcus from the Ruminococcaceae family (r = 0.880, *p* = 0.0206). Beta-Muricholic acid showed a high correlation with Alistipes (r = 0.943, *p* = 0.0167). Gamma-Linolenic acid and alpha-Linolenic acid also showed moderate positive correlations with Alistipes and Odoribacter. Eicosapentaenoic acid showed negative correlations with Ruminococcus and Ruthenibacterium. Indole-3-propionic acid correlated with Eubacterium (Figure 6A). Between the model group and the 12-week reversal group, Blautia showed significant negative correlations with 15-Deoxy-PGJ2, all-trans-5,6-Epoxyretinoic acid, alpha-Linolenic acid, Hyodeoxycholic acid, and Linoleic acid. Ursodeoxycholic acid and beclomethasone propionate showed positive correlations with Roseburia (r = 0.886, *p* = 0.0333). 15-Deoxy-PGJ2 and all-trans-5,6-Epoxyretinoic acid also showed varying degrees of positive correlation with Adlercreutzia and Alloprevotella (Figure 6B). Comparing the 4-week reversal group with the 12-week reversal group, linoleic acid was closely associated with Lawsonibacter, Roseburia, and Ruthenibacterium. Additionally, 15-Deoxy-PGJ2, all-trans-5,6-Epoxyretinoic acid, and Indoleacrylic acid showed correlations with Agathobaculum, Choladousia, and Massilioclostridium (Figure 6C).

In summary, correlation analysis indicates that the liver–gut axis undergoes continuous evolution during the reversal process: early stages are primarily characterized by associations between basal metabolites and the microbiota, while later stages develop into a complex regulatory system integrating hepatoprotective bile acids, pro-resolution lipid mediators, and specific functional microbiota.

## 4. Discussion

This study integrated non-targeted metabolomics data with 16S rRNA gene sequencing data to reveal the dynamic changes in gut metabolites and microbial community structure during the spontaneous reversal of liver fibrosis in mice. We first identified key metabolites and focused on enriched metabolic pathways during the reversal process. Subsequent 16S rRNA gene sequencing revealed that the reversal of liver fibrosis is not driven by the overall α-diversity of the gut microbiota, but rather accompanies significant restructuring of community composition (β-diversity) and activation of specific metabolic pathways. More importantly, we observed a close association between key bacterial groups and metabolic products, providing new evidence for elucidating the underlying mechanisms of liver fibrosis reversal.

Our non-targeted metabolomics analysis, combined with heatmap visualization, clearly revealed a set of characteristic metabolites undergoing significant changes during the reversal of liver fibrosis. Gamma-linolenic acid (GLA) and alpha-linolenic acid (ALA) exhibited the highest expression levels in the model group and decreased significantly during the reversal process. GLA and ALA serve as crucial precursors for synthesizing anti-inflammatory lipid mediators (e.g., prostaglandins and regurgitins derived from the omega-6 and omega-3 series) [13,14,15]. Fibrotic states may be associated with the abnormal accumulation of lipid precursors. Therefore, their reduction during reversal likely correlates with reduced hepatic inflammatory responses, potentially explaining one mechanism underlying fibrosis reversal. Additionally, we identified multiple specific metabolites exhibiting significant changes during reversal, which synergistically drive liver fibrosis reversal at multiple levels. Observations revealed that compared to the model group, PE(14:0/16:1(9Z)) showed restorative changes in the reversal groups. Previous studies indicate that PE alleviates liver injury, regulates intestinal bile acid metabolism, and protects intestinal health in mice [16]. Phospholipids are major components of cell membranes, and alterations in their composition directly affect membrane fluidity, receptor function, and cellular signaling. PE(14:0/16:1(9Z)), a key phospholipid in cell membranes, participates in membrane synthesis, degradation, and remodeling [17]. Its elevated levels during reversal suggest that successful fibrosis reversal may depend on specific phospholipid metabolic reprogramming to support membrane structural regeneration and functional recovery. Lysophosphatidylcholine, a pro-inflammatory lipid mediator, exhibits elevated levels during fibrosis but decreases during reversal, potentially reflecting inflammatory resolution and advancing tissue repair [18,19,,20].

Regarding gut microbiota, we found that during the reversal of liver fibrosis, α-diversity (Chao1, Shannon index, etc.) showed no significant changes. However, β-diversity analysis revealed significant differences in microbial community structure among the three groups. This result suggests that the reversal of liver fibrosis may be more closely associated with the rise and fall of specific functional microbial communities rather than alterations in overall community size. Some recognized beneficial bacteria also exhibit distinct patterns of change. For example, Akkermansia showed significantly higher relative abundance in the 12-week reversal group compared to the model group and the 4-week reversal group. Akkermansia has been extensively reported to improve intestinal barrier function and regulate mucous layer thickness, with increased abundance typically associated with enhanced metabolic health [21,22,23]. Additionally, the relative abundance of Lachnospiraceae peaked in the 4-week reversal group. This family includes multiple genera producing short-chain fatty acids (such as butyrate). Butyrate serves as a vital energy source for colonic epithelial cells and possesses multiple physiological functions, including anti-inflammatory effects and maintenance of intestinal barrier integrity [24,25,26]. The restoration of these functionally distinct beneficial bacteria may collectively establish an intestinal microenvironment conducive to liver repair. However, as this study is based solely on correlation analysis, it cannot determine whether these changes in the microbiota are drivers of reversal or merely phenomena that accompany the recovery of the liver and intestinal environments. In fact, there may be bidirectional interactions between the gut microbiota and liver disease: dysbiosis can exacerbate liver fibrosis, while the recovery from liver injury may similarly reshape the microbiota structure by altering bile acid secretion, intestinal permeability, and the local immune environment. This bidirectional nature makes it difficult for a single cross-sectional correlation analysis to distinguish the direction of causality. Sahu et al. noted that although numerous studies have described the association between microbiota changes and liver disease, there remain limitations in establishing causality [27]. Furthermore, Li et al. observed that the relationship between increased beneficial microbiota and improved liver fibrosis may represent a bidirectional, interactive cycle rather than a unidirectional causal relationship [28]. Therefore, future studies should utilize fecal microbiota transplantation or strain-specific colonization experiments to further validate the causal relationship between the potentially protective bacterial genera identified in this study and the reversal of liver fibrosis.

In exploring mechanisms for reversing liver fibrosis, the role of the “liver–gut axis” has become increasingly prominent. The liver and gut are closely interconnected via the portal vein, biliary tract, and circulatory system. The gut microbiota and its metabolites act as key participants, influencing the physiological and pathological states of the liver through multiple pathways, including immune regulation, barrier function, and metabolism [29,30,31]. Research indicates that dysbiosis of the gut microbiota can promote bacterial translocation, increase the risk of endotoxemia, and activate hepatic immune responses, thereby exacerbating liver injury and fibrosis [32,33,34]. Conversely, maintaining gut microbiota homeostasis may reverse the progression of liver fibrosis.

The core finding of this study lies in the preliminary establishment of a potential mechanism chain linking “gut microbiota-metabolites-reversal of liver fibrosis” through multi-omics integration analysis. We found that the reversal of liver fibrosis is not merely a process of increasing beneficial bacteria and reducing harmful bacteria, but rather a multi-stage dynamic process accompanied by intense succession within the microbiota and the reconstruction of metabolic networks. In the early stages of reversal, characterized by the proliferation of short-chain fatty acid-producing bacteria such as the family Ruminococcaceae and the activation of sphingolipid metabolism, this may be associated with early signals for anti-inflammation and promoting the quiescence of hepatic stellate cells. In the later stages of reversal, gut microbiota structure undergoes further adjustment. Akkermansia, a gut bacterium with probiotic potential, shows a significant increase in abundance during improved liver fibrosis, potentially aiding in gut barrier repair. Conversely, abnormal proliferation of Escherichia coli may reflect ongoing gut microbiome restructuring at this stage, with long-term consequences requiring further evaluation. Concurrently, the host’s metabolic phenotype undergoes corresponding shifts, extending from early lipid metabolism adjustments to later amino acid and bile acid metabolic remodeling. The predicted functional profiles of the gut microbiota from PICRUSt2 showed enrichment of amino acid and energy metabolism during the initial reversal phase, which temporally aligned with host pathways identified in our metabolomics, such as sphingolipid metabolism, α-linolenic acid metabolism and vitamin metabolism. This cross-omics synchrony during the initial reversal phase suggests that functional remodeling of the gut microbiota closely coordinates with systemic metabolic repair in the host, potentially forming a metabolic basis for fibrosis reversal driven by the gut-liver axis. However, the predicted differences between the 4-week and 12-week reversal groups were not centered on core metabolic pathways, but rather on pathways related to the nervous system, viral infectious diseases, specific cancers, and the circulatory system. We speculate that this temporal shift may reflect a change in the inferred functional potential of the gut microbiota, possibly from early metabolic support toward pathways more closely associated with immune and systemic physiological regulation. Furthermore, the enrichment of endocrine system and parasitic infection-related pathways between the model group and the 12-week reversal group offers a potential indication that long-term restoration of gut microbiota function may be associated with recovery of endocrine homeostasis and suppression of infection-associated microbial functions.

These changes are tightly interconnected via the liver–gut axis, where gut microbiota may enter the portal venous circulation through their metabolites, directly or indirectly influencing hepatic inflammation, oxidative stress, and the activation state of hepatic stellate cells. Our data provide direct evidence from the gut contents, microbiome, and metabolome supporting this mechanism.

This study also has several limitations. First, we employed non-targeted metabolomics to detect metabolite changes; however, in-depth analysis of specific metabolic pathways still requires validation through targeted metabolomics, such as precise quantification of bile acid profiles. This would facilitate a deeper understanding of the specific roles these metabolites play in fibrosis reversal. Second, this study primarily revealed correlations between microbiota and metabolites rather than causal relationships. Future studies utilizing germ-free animal models for fecal microbiota transplantation experiments could help validate the causal role of specific microbiota and their metabolites in reversing liver fibrosis. Furthermore, our findings are based solely on a single mouse spontaneous reversal model. Validation using independent fibrosis reversal models (e.g., dietary models) or clinical patient samples before and after effective anti-fibrotic therapy will be necessary to confirm the translational relevance of the identified metabolite–microbiota associations. Additionally, larger-scale cohort studies are needed to validate the conclusions drawn from this study.

## 5. Conclusions

In summary, this study employs multi-omics integration analysis to reveal the dynamic characteristics of metabolic remodeling during spontaneous reversal of liver fibrosis and its intrinsic connection with gut microbiota restructuring. These findings not only provide new insights into the metabolic mechanisms underlying fibrosis reversal but also offer crucial theoretical foundations for developing metabolic regulation-based therapeutic strategies for liver fibrosis.

## Data Availability

The original contributions presented in this study are included in the article; further inquiries can be directed to the corresponding author.

## Supplementary Materials

The following supporting information can be downloaded at: https://www.mdpi.com/article/10.3390/metabo16060424/s1, Table S1: 23 differentially expressed metabolites identified based on expression levels between any two of the three groups; Table S2: Inter-group β-diversity analysis; Table S3: Inter-group β-diversity analysis.

## Abbreviations

16S rRNA	16S ribosomal RNA
ECM	Extracellular matrix
NAFLD	Non-alcoholic fatty liver disease
CCl_4_	Carbon Tetrachloride
QC	Quality control
UPLC	Ultra-high-performance liquid chromatography
LC	Liquid Chromatography
PCA	Principal component analysis
OPLS-DA	Orthogonal partial least squares discriminant analysis
KEGG	Kyoto Encyclopedia of Genes and Genomes
ANOSIM	Analysis of similarities
GLA	Gamma-linolenic acid
ALA	Alpha-linolenic acid
